# Acute Effect of Hypervolemic Hemodilution on Retrobulbar Hemodynamics in Anterior Ischemic Optic Neuropathy

**DOI:** 10.1155/2018/4756313

**Published:** 2018-02-06

**Authors:** Marion Bienert, Niklas Plange, Andreas Remky, Kay Oliver Arend, David Kuerten

**Affiliations:** ^1^Department of Ophthalmology, Uniklinik RWTH Aachen, Aachen, Germany; ^2^Augen-Beleg-Klinik, Krankenhaus Barmherzige Brüder, Regensburg, Germany; ^3^Augenzentrum Annapark, Alsdorf, Germany

## Abstract

**Purpose:**

Ischemic ocular disorders may be treated by hypervolemic hemodilution. The presumed therapeutic benefit is based on a volume effect and improved rheological factors. The aim was to investigate the acute effect of intravenous hydroxyethyl starch on retrobulbar hemodynamics in patients with nonarteritic anterior ischemic optic neuropathy (NAION).

**Methods:**

24 patients with acute NAION were included. Retrobulbar hemodynamics were measured using color Doppler imaging before and 15 min after intravenous infusion of 250 cc 10% hydroxyethyl starch (HES). Peak systolic velocity (PSV), end diastolic velocity (EDV), and Pourcelot's resistive index (RI) were measured in the ophthalmic artery (OA), central retinal artery (CRA), and short posterior ciliary arteries (PCAs).

**Results:**

After infusion of HES blood flow velocities significantly increased in the CRA (PSV from 7.53 ± 2.33 to 8.32 ± 2.51  (*p* < 0.001); EDV from 2.16 ± 0.56 to 2.34 ± 0.55  (*p* < 0.05)) and in the PCAs (PSV from 7.18 ± 1.62 to 7.56 ± 1.55  (*p* < 0.01); EDV from 2.48 ± 0.55 to 2.66 ± 0.6 cm/sec (*p* < 0.01)). The RI of all retrobulbar vessels remained unaffected. Blood pressure and heart rate remained unchanged.

**Conclusions:**

Hypervolemic hemodilution has an acute effect on blood flow velocities in the CRA and PCAs in NAION patients. Increased blood flow in the arteries supplying the optic nerve head may lead to a better perfusion in NAION patients. This trial is registered with DRKS00012603.

## 1. Introduction

The etiology of nonarteritic anterior ischemic optic neuropathy (NAION) is believed to be multifactorial resulting in an acute hypoperfusion of short posterior ciliary arteries supplying the optic nerve [1, 2]. The pathogenic mechanisms triggering an acute ischemic lesion of the optic nerve head encompass various vascular risk factors and an acute incident of hypoperfusion: for example, nocturnal arterial hypotension [3], aggressive antihypertensive therapy [4], or hypotension after dialysis [5]. It was reported that NAION can occur even if the perfusion in only one PCA is transiently disturbed [6]. Furthermore, it is believed that the PCAs are often reperfused after the initial occlusion, as no thrombosis in the PCAs in NAION patients was found in two histopathological studies [7, 8].

Several studies investigated circulatory abnormalities in ocular blood flow in patients with NAION. Prolonged arteriovenous passage times and significantly delayed filling of the optic nerve head capillaries in fluorescein angiography in NAION patients were reported [9, 10]. Furthermore, patients with NAION showed decreased velocities in the capillaries of the optic nerve head measured by laser Doppler velocimetry [11]. Impaired retrobulbar hemodynamics of patients with NAION have been shown in previous studies by means of color Doppler imaging (CDI) [12–15]. We previously presented data on reduced blood flow velocities in the CRA and nasal PCAs in patients with acute NAION compared with age-matched healthy control eyes [15].

In NAION, an acute hypoperfusion of short posterior ciliary arteries results in optic nerve head ischemia leading to a sudden painless visual field defect with an optic disc edema. Multiple medical and surgical treatment options have been investigated, including levodopa [16], hyperbaric oxygen [17, 18], hemorheological intervention [19, 20], and optic nerve sheath decompression [12, 21, 22], but no proven effective treatment is currently available. Most commonly corticosteroids are administered systemically [23].

The effect of hemodilution in NAION using hydroxyethyl starch infusion was investigated in a prospective randomized study in 1991. The initial results showed an improvement of visual acuity three months after isovolemic hemodilution in six of eleven patients in comparison to one of ten patients in the control group (*p* = 0.024) [20]. No long-term results were published from this study. Another prospective, uncontrolled study found a significantly reduced arteriovenous passage time, a lower plasma viscosity, and an improvement of central vision by 2 or more lines in seven of 22 patients 10 days after hemodilution with hydroxyethyl starch [24]. After nineteen months, 50% of the patients showed a better visual acuity and 36% had a better visual field compared to baseline [25]. In a retrospective study with 24 patients, isovolemic hemodilution had no significant long-term effect on visual acuity and visual fields after 24 months, but it showed a nonsignificant tendency to lower recurrence rate of NAION in the affected eye [19].

Even though placebo-controlled long-term studies of hemodilution therapy in NAION are missing, hypervolemic hemodilution using hydroxyethyl starch might be beneficial in the acute treatment resulting in improved blood flow in the optic nerve. In our department, the initial infusion of hydroxyethyl starch is immediately given after the diagnosis NAION has been confirmed as a standard clinical regimen followed by isovolemic hemodilution for 3–5 days. The present study investigates the acute effect of the initial hydroxyethyl starch infusion on retrobulbar hemodynamics in patients with NAION.

## 2. Materials and Methods

### 2.1. Patients

Twenty-four consecutive patients (mean age: 67 ± 9 years) with nonarteritic unilateral AION were enrolled in this study. Patients were included if they presented a pathognomonic acute painless visual field loss and an optic disc edema without evidence of other neurological or ocular diseases. All patients were studied within 4 weeks of onset of visual field loss (mean latency: 7 days). The trial was registered with DRKS00012603.

Patients with high erythrocyte sedimentation rate (ESR > 35/65 mm/hr) and increased C-reactive protein (CRP > 10 mg/l) or with clinical features of giant cell arteritis (headache or paresthesia next to the superficial temporal artery) were excluded from this study. Furthermore, exclusion criteria were contraindications to hydroxyethyl starch infusion like arterial blood pressure higher than 170/90 mmHg, chronic renal insufficiency, or known allergy against hydroxyethyl starch.

Demographic and clinical data of the patients are given in Table 1.

### 2.2. Methods

All patients had a detailed ophthalmological examination and visual field testing with the Goldmann perimeter or the Humphrey Field Analyzer (Model 750, Humphrey-Zeiss, San Leandro, California, USA) using a white-on-white 30-2 program and color Doppler imaging examination (CDI). The visual acuity was given in logMAR. Patient's history was explored with special interest in cardiovascular risk factors (i.e., diagnosis of treated hypertension, diabetes, atherosclerosis, or nicotine abuse; atherosclerosis included a history of myocardial infarction, stroke, or stenosis of the carotid artery).

The eye, gender, and age of the patients and latency between onset of visual field loss and examination were registered. ESR, C-reactive protein, and creatinine were determined.

The retrobulbar hemodynamics were measured before and 15 min after an intravenous infusion (antecubital vein) of 250 cc 10% hydroxyethyl starch (molecular weight: 200.000/degree of substitution 0.5) within 30 min. The same testing conditions for the CDI measurements existed prior to and after the infusion. The investigation followed the tenets of the Declaration of Helsinki and was approved by the local ethics committee.

Retrobulbar blood flow velocities of the ophthalmic artery (OA), the central retinal artery (CRA), and the temporal and nasal posterior ciliary arteries (PCAs) were determined by CDI by the same experienced investigator (MB) [13]. A 7.5 MHz linear probe (Sonoline Sienna Siemens, Munich, Germany) was applied to a closed eyelid using a coupling gel. Samples of pulsed-Doppler signal from within a 1.2 × 1.2 mm sample volume were analyzed to calculate blood velocities. The Doppler-shifted spectral waveforms of each vessel were recorded over a period of more than five seconds. Over that period constant samples were required before velocities were measured. In each vessel, peak systolic velocity (PSV) and end diastolic velocity (EDV) were determined from the Doppler-shifted spectral waveform. In addition, Pourcelot's resistive index (RI), a measure of peripheral vascular resistance, was calculated as follows: RI = (PSV – EDV)/PSV.

The OA was examined approximately 25 mm behind the globe at the straighter portion of the vessel in the nasal orbit to obtain the most reliable results [24]. The CRA was measured approximately 10 mm behind the optic nerve head within the retrolaminar portion of the optic nerve. The PCAs were detected 10–20 mm behind the globe, where they commence as nasal and temporal trunks to the optic nerve before forming multiple branches surrounding the optic nerve in its retrobulbar portion [23]. The hemodynamic parameters of the PCAs are the means of the nasal and temporal PCAs.

Heart rate and blood pressure were determined by sphygmomanometry in a sitting position after a rest of 5 minutes before and 15 minutes after the infusion of hydroxyethyl starch (Poet Te plus, Criticare Systems, Wisconsin, USA). The mean arterial blood pressure was calculated as follows: mean arterial blood pressure = diastolic blood pressure + 1/3 (systolic blood pressure − diastolic blood pressure).

### 2.3. Statistical Methods

For the statistical analysis of this study, a paired *t*-test was applied for comparisons before and after hydroxyethyl starch. Correlations were calculated by Fisher's transformation. Fisher's exact test was used in the analysis of contingency tables. In all analyses, *p* < 0.05 was regarded as statistically significant.

## 3. Results

Blood flow velocities and resistive indices of the patients with acute NAION before and after the infusion of 250 cc 10% hydroxyethyl starch are summarized in Table 2.

**Figure 1:**
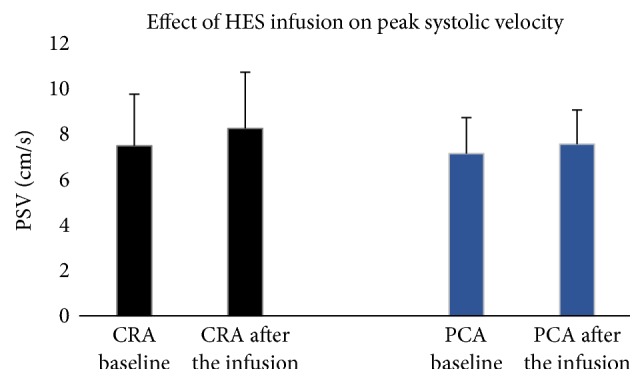
Effect of the infusion of 250 cc hydroxyethyl starch (HES) on peak systolic velocity (PSV) in the central retinal artery (CRA) and posterior ciliary artery (PCA).

**Figure 2:**
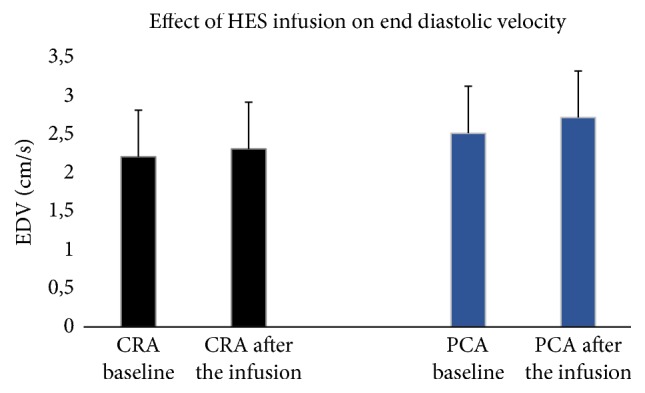
Effect of the infusion of 250 cc hydroxyethyl starch (HES) on end diastolic velocity (EDV) in the central retinal artery (CRA) and posterior ciliary artery (PCA).

After infusion of hydroxyethyl starch the peak systolic and end diastolic blood flow velocities significantly increased in the CRA and PCAs compared to the pretreatment velocities. In CRA, the PSV increased from 7.5 ± 2.3 to 8.3 ± 2.5 cm/sec (*p* < 0.001) and the EDV from 2.2 ± 0.6 to 2.3 ± 0.6 cm/sec (*p* < 0.05). In PCAs, the PSV rose from 7.2 ± 1.6 to 7.6 ± 1.6 cm/sec (*p* < 0.01) and the EDV from 2.5 ± 0.6 to 2.7 ± 0.6 cm/sec (*p* < 0.01). Please refer to Figures 1 and 2 for visualization.

We found no correlation between the relative changes of retrobulbar blood flow velocities. However, there was a significant negative correlation between the relative change and the pretreatment velocity of the EDV in CRA (*r* = −0.53, *p* < 0.01) and of the PSV in PCAs (*r* = −0.43, *p* < 0.05). The PSV in the CRA and the EDV in the PCAs showed no correlation between the relative change and the baseline velocity. No correlation between the relative changes and time to treatment was recorded.

For further analysis of changing velocities, contingency tables were calculated with two groups. The first group with a relative change of velocity more than the median and the second group with a relative change less than or equal to the median were determined. In the CRA, all patients with a relative change of PSV above the median (>9.35%) showed a relative change of EDV above the median (>5.61%). Fisher's exact *p* value was <0.0001. In the PCA, eight of twelve patients with a relative change of PSV above the median (>3.33%) showed a relative change of EDV above the median (>4.94%). Fisher's exact *p* value was not significant (*p* = 0.22).

The RI of all retrobulbar vessels remained unaffected. The OA showed no significant changes in any hemodynamic parameter after hypervolemic hemodilution. Blood pressure and heart rate remained unchanged (Table 3).

## 4. Discussion

After hypervolemic hemodilution using hydroxyethyl starch, blood flow velocities in the CRA and the PCAs are significantly increased in acute NAION. Higher blood flow velocities measured by CDI may be interpreted as either an increase in blood flow, a lower peripheral resistance, or a decrease of vessel diameter. We did not find any changes of the resistive indices of the retrobulbar vessels after hydroxyethyl starch; therefore an increase in vessel resistance seems unlikely. Spencer et al. showed that simultaneous changes of the peak systolic velocity and end diastolic velocity of the CRA without changes of resistive indices may be interpreted as altered volumetric flow in this vessel [26]. Therefore, hypervolemic hemodilution using hydroxyethyl starch appears to result in an increase in blood flow in the vessels supplying the optic nerve in patients with NAION. This phenomenon might be due to the volume effect of hydroxyethyl starch [27]. In healthy volunteers, a significantly elevated blood flow in the common carotid artery could be assessed three hours after infusion of 500 ml of 10% medium molecular weight hydroxyethyl starch (200,000/0.62) without any changes of blood pressure [28]. In our study, blood pressure remained unchanged as well. Therefore, a direct influence of blood pressure changes on retrobulbar flow velocities seems unlikely.

An increase in flow velocities after hypervolemic hemodilution, presumed as a higher volumetric flow in the retrobulbar vessels, might be due to more than a volume effect alone. Patients with NAION showed a higher plasma viscosity in comparison to healthy controls [29] and HES leads to an improvement of rheological factors like plasma viscosity and erythrocyte aggregation [30]. 10 days of hemodilution with hydroxyethyl starch resulted in lower plasma viscosity and shortening of arteriovenous passage time in patients with NAION [24]. We found in our study a relation between the peak systolic velocity and end diastolic velocity concerning the extent of the acceleration after hydroxyethyl starch. In the CRA, patients with a more profound increase in the PSV also showed similar increases in the EDV. The same tendency was measured in the PCAs but was not found to be significant. This might be due to the higher variability of CDI measurements in the PCAs [31]. Furthermore, overall significant differences were only recorded for the CRA and PCAs. The CDI parameters recorded in the OA remained unchanged after HES infusion. First the OA is not affected in NAION; and therefore high blood flow velocities can be recorded, and vascular autoregulation is presumed to be intact. Furthermore, the changes in ocular perfusion after HES infusion remain minimal and do not become statistically significant if the baseline values are high. In the CRA and the PCAs significantly reduced blood flow can be recorded in NAION patients; therefore even small changes after HES infusion are able to lead to statistically significant increases in the EDV and PSV as recorded in our study. The overall reduced blood flow in the CRA is believed to be due to the edema of the optic nerve head surrounding the CRA and hypoperfusion/occlusion in the PCAs is the main cause of AION.

Lately, HES solutions were critically reviewed and the marketing authorizations for the EU were withdrawn [32]. Although HES solutions achieve better resuscitation of the microcirculation in humans than normal saline solutions [33], the administration of HES in critically ill patients was accompanied by a greater risk of kidney injury [34, 35]. The American FDA decided accordingly and recommended that HES products should not be used in critically ill patients or in those with preexisting renal dysfunction [36]. Alternatives for HES as colloid volume expanders also lack sufficient safety data [37] and volume expansion solely via crystalloid is not as effective.

Overall, it is not known if the use of HES is dangerous in NAION patients to date. The administration appears to be safe in these patients, who in most cases are not critically ill and do not end up in an intensive care unit; however the administration in patients with preexisting renal dysfunction and renal diseases appears questionable.

In this study, we present increased blood flow velocities in the PCAs and CRA after hypervolemic hemodilution in patients with NAION. Negative correlations between the pretreatment velocity and the relative change were found in the EDV of CRA and in the PSV of PCAs, suggesting that patients with low velocities at baseline showed a higher increase of flow velocities compared to patients with higher pretreatment velocities. The acceleration of flow velocities by HES in the vessels with reduced blood flow might represent an improvement of the acute circulatory disorder in NAION.

However, similar results of improved perfusion parameters were initially reported for optic nerve sheath fenestration (ONSF) [12]. Nevertheless, the large-scale IONDT trial showed no clinical benefit of the intervention and that it is accompanied by an even higher risk of severe visual acuity deterioration [21]. Therefore, further studies seem necessary if an increase of retrobulbar flow velocities is linked to a better outcome in visual function in patients with NAION treated with HES. Increasing ocular blood might be a beneficial therapeutic approach in particular due to the lack of a conclusive general therapeutic regime for NAION [23, 24].

A limitation of this study is the different latencies between the onset of symptoms of NAION and the initiation of hemodilution. However, we found no correlation between the latency and the relative change of the velocities after treatment. Another limitation is a possible influence of antihypertensive drugs and systemic diseases on retrobulbar hemodynamics. Furthermore, the IOP was not measured after HES infusion; therefore, one can only speculate that the ocular perfusion pressure remained unaffected by the infusion. The systolic and diastolic blood pressure values remained stable after HES infusion.

In conclusion, this study showed an acceleration of the peak systolic velocity and end diastolic velocity in the CRAs and PCAs after infusion of hydroxyethyl starch. An improvement of the retrobulbar circulation might be beneficial in the treatment of NAION.

## Figures and Tables

**Table 1:** Demographic and clinical data of the 24 patients with NAION.

Gender male/female (*n*)	15/9
Eye: right/left (*n*)	10/14
Mean age ± standard deviation (years)	67 ± 9
Visual acuity ± standard deviation (logMAR)	0.58 ± 0.56
Mean defect (Humphrey 30-2) ± standard deviation (dB)	−15.7 ± 7.5
Latency ± standard deviation (days)	7.1 ± 6.7
CRP (mg/l) < 5/5–10 (*n*)	14/9
Hematocrit ± standard deviation	0.44 ± 0.04
Arterial hypertension on therapy (*n*)	11
Atherosclerosis (*n*)	10
Diabetes mellitus (*n*)	4
Current smokers (*n*)	7

**Table 2:** Blood flow velocities (PSV: peak systolic velocity and EDV: end diastolic velocity) and resistive indices (RI) of the retrobulbar vessels (ophthalmic artery (OA), central retinal artery (CRA), and posterior ciliary arteries (PCA)) in patients with NAION before and after infusion of 250 cc 10% hydroxyethyl starch (mean ± standard deviation is given).

	Before	After therapy	*p* value	Relative change in %
OA PSV (cm/s)	34.5 ± 8.1	36.4 ± 9.4	0.13	6.0 ± 19.4
OA EDV (cm/s)	7.0 ± 2.5	7.3 ± 2.8	0.52	8.9 ± 35.2
OA RI	0.8 ± 0.06	0.8 ± 0.06	0.89	0.8 ± 7.7
*CRA PSV (cm/s)*	7.5 ± 2.3	8.3 ± 2.5	*0.0006*	11.4 ± 15.8
*CRA EDV (cm/s)*	2.2 ± 0.6	2.3 ± 0.6	*0.03*	11.1 ± 23.3
CRA RI	0.7 ± 0.08	0.71 ± 0.07	0.66	1.1 ± 8.1
*PCA PSV (cm/s)*	7.2 ± 1.6	7.6 ± 1.5	*0.009*	6.3 ± 10.7
*PCA EDV (cm/s)*	2.5 ± 0.6	2.7 ± 0.6	*0.003*	7.8 ± 11.5
PCA RI	0.65 ± 0.06	0.65 ± 0.05	0.72	−0,2 ± 7.0

OA: ophthalmic artery; CRA: central retinal artery; PCA: posterior ciliary artery; PSV: peak systolic velocity; EDV: end diastolic velocity; RI: resistive index.

**Table 3:** Blood pressure and heart rate of the patients with NAION before and after infusion of 250 cc 10% hydroxyethyl starch (mean ± standard deviation is given).

	Before	After therapy	*p* value
Systolic blood pressure (mmHg)	149 ± 18	147 ± 17	0.76
Diastolic blood pressure (mmHg)	83 ± 11	83 ± 12	0.78
Mean arterial pressure (mmHg)	105 ± 10	104 ± 12	0.71
Heart rate (beats/min)	74 ± 11	75 ± 9	0.56
